# Change in Daily Steps and Self-efficacy of Online Interactive Exercise Classes for Community-dwelling Older Adults in Japan: A Preliminary Study

**DOI:** 10.5195/ijt.2022.6492

**Published:** 2022-12-13

**Authors:** Mami Ishizuka-Inoue, Kanako Shimoura, Reika Yamashita, Miyu Onishi, Takanobu Saito, Momoko Nagai-Tanima, Tomoki Aoyama

**Affiliations:** Department of Human Health Sciences, Graduate School of Medicine, Kyoto University, Kyoto City, Kyoto, Japan

**Keywords:** Elderly, Exercise programs, Preliminary study, Self-efficacy, Telehealth

## Abstract

**Aims:**

This study aimed to (1) examine the feasibility of an online interactive exercise class for community-dwelling older adults and (2) preliminarily examine changes in physical activity and self-efficacy.

**Methods:**

Participants were 25 community-dwelling older adults aged 65 years or older, but due to 5 dropouts, the final number of participants for analysis was 20 (mean age 76.9 ± 5.7 years). The intervention program was conducted for 40 minutes each session, twice a week for four consecutive weeks, using the LINE group call (LINE Corporation, Japan). An online questionnaire was used to assess participant characteristics, modified Fall Efficacy Scale score, modified Gait Efficacy Scale (m-GES) score, self-rated health, and daily steps, which were compared pre- and post-intervention using the Wilcoxon signed-rank sum and chi-square tests.

**Results:**

The Wilcoxon signed-rank sum test showed significant improvement in the m-GES score and daily steps. The chi-square test showed that self-rated health was significantly greater in the maintenance/increase group.

**Conclusions:**

Online interactive exercise classes are feasible for community-dwelling older individuals. These results also suggest the possibility of using telehealth to improve physical activity and self-efficacy.

In Japan, physical fitness tests and classroom-based exercise intervention programs organized by local governments and nursing care facilities are recommended as preventive care programs for community-dwelling older adults (Ministry of Health, Labour and Welfare, 2022a). Classroom-based exercise intervention programs that involve strength and balance training have been reported to improve physical activity, exercise-related self-efficacy, and self-rated health, and are effective to a certain extent (Barenfeld et al., 2018; Nishiguch et al., 2015; Suzuki et al., 2004). Physical activity, such as exercise and walking, reduces the risk of falls and mortality (Hakim et al., 1998; Province et al., 1995). Additionally, 25%–45% of older adults have limited physical activity due to the decreased exercise-related self-efficacy (Fletcher et al., 2004; Murphy et al., 2002; Tinetti et al., 1994). Therefore, it is important to maintain and improve self-efficacy, and encourage physical activity through the implementation of exercise intervention programs.

However, according to the Ministry of Health, Labour and Welfare's report, the content and frequency of care prevention programs vary across regions (Ministry of Health, Labour and Welfare, 2020). Some older people tend to be confined and do not participate in community activities due to road traffic and climatic conditions (Ministry of Health, Labour and Welfare, 2022a). A comparison of the healthy life expectancy of people living in each region according to regional classification by administrative division in Japan reported that the largest difference in healthy life expectancy was 2.79 years for men and 2.95 years for women (Ministry of Health, Labour and Welfare, 2013), which may be due in part to the differences in approaches to long-term care prevention projects. Because of this, Japan is considered a health disparity society (Ministry of Health, Labour and Welfare, 2013).

Health disparities refer to the differences in health status by region and socioeconomic status (Ministry of Health, Labour and Welfare, 2013). *Health Japan 21*, which outlines the basic policies to promote the health of people by the government, is aimed at eliminating health disparities, and each municipality is reviewing its policies for health promotion and disease prevention (Ministry of Health, Labour and Welfare, 2013).

One approach to eliminating health disparities is through the use of online interventions. Online interventions would attenuate regional differences because people can participate anywhere. In a study conducted in Turkey, community-dwelling older individuals were asked to watch online videos and perform flexibility exercises for four weeks; as a result, depression, fear of falling, and balance function were reportedly improved (Tekin et al., 2022). In Japan, many local governments have distributed exercise videos for older adults on YouTube to encourage them to exercise (Ministry of Health, Labour and Welfare, 2022b), but the effectiveness of this program has not been verified. Moreover, a previous study reported that the same type of exercise is more effective in preventing falls when performed by group rather than by a solitary individual (Hayashi et al., 2018). Therefore, a group interactive program may be more effective compared with video streaming, in which exercises are performed individually. Previous researchers examining online interactive exercise classes have conducted feasibility studies on patients with Parkinson's disease and cancer and reported feasibility and improved physical activity (Dennett et al., 2021; Flynn et al., 2021). However, no existing study has examined the feasibility of an online interactive exercise class in community-dwelling older adults. Therefore, this study aimed to (1) examine the feasibility of an online interactive exercise class in community-dwelling older adults and (2) preliminarily examine changes in physical activity and self-efficacy.

## Methods

### Participants

This one-arm, pre- and post-comparison study targeted community-dwelling older individuals 65 years or older. Participants were recruited from an unspecified number of people by posters and flyers at local community centers and welfare centers. Individuals (1) aged 65 years or older, (2) who were not certified for long-term care or support under Japan's long-term care insurance system, (3) who were not using a walking aid daily, and (4) with no exercise restrictions imposed by their primary doctors, were eligible for this study. The mobile messenger application LINE (LINE Corporation, Japan) was used throughout the application, intervention, and evaluation period. LINE is an application used by 80∼90% of the Japanese population, allowing individual and group chats and video calls. The participants were registered as friends with the official LINE account used in this study utilizing the QR code posted on the flyer. At the time of application, an online questionnaire was used to confirm that the participants met the selection criteria. The study participants were individually briefed regarding the details of the study via videoconference prior to its initiation, and informed consent was obtained. A total of 25 applicants were included in the study. This study was approved by the Medical Ethics Committee of Kyoto University (C1529-1).

### Intervention Program

The intervention program lasted 40 minutes per session and was conducted twice a week for four consecutive weeks. The study was performed from October to November 2021, and all participants were asked to select a schedule during which they could participate in the program for four consecutive weeks. The intervention period was in two patterns: (i) from October 3 to October 30, 2021, or (ii) from October 31 to November 27, 2021. Three time slots were set up for each intervention day, and participants were asked to choose the time of day they would like to participate. The intervention days were the same dates for all groups. The participants were randomly divided into groups of 3–7 people according to their preferred period and time, and a LINE talk group was created that included a study conductor. The intervention program was implemented in each group by conducting a LINE video call with a two-way communication between the study conductor and the participants. LINE video calls were displayed on one screen simultaneously for up to nine participants when using a tablet device, so the research conductor was able to understand the movements and voices of all participants at the same time, even for the largest group of seven participants. Participants were instructed to make the screen of the research conductor larger than the other members to facilitate understanding of the exercise.

**Figure 1:**
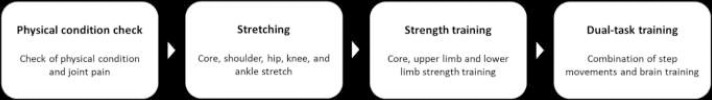
Intervention Program Content

The content of the program was devised based on the Ministry of Health, Labour and Welfare's manual on care prevention (Ministry of Health, Labour and Welfare, 2022a). The program consisted of a physical condition check (5 min), stretching (5 min), strength training (10 min), and dual-task training combining step movements and cognitive training (Nishiguch et al., 2015) (20 min). The details are shown in Figure 1. Stretching and strength training were performed with the participant in a seated position. Dual-task training was performed in sitting and standing positions.

**Figure 2:**
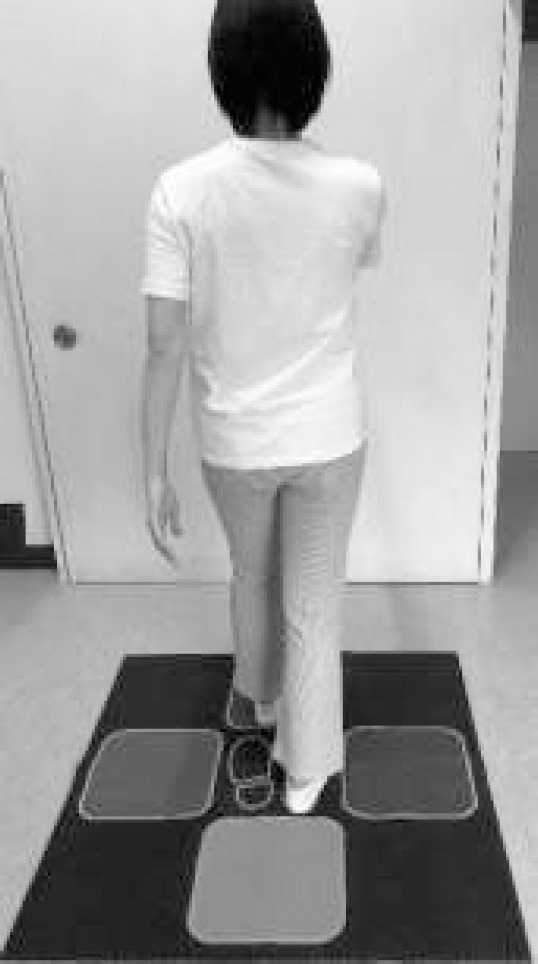
Exercise Mat

The instructors were required to be licensed physical therapists. Each group was evaluated on their understanding of the exercises and fatigue levels, and the exercise content was adjusted accordingly. The participants were provided exercise mats designed in colors and shapes to help convey instructions and prevent slipping on the floor surface (Figure 2).

### Assessment of Feasibility

To assess the feasibility of the program, the withdrawal and attendance rates were evaluated throughout the program. The participants were allowed to withdraw from the study at any time and asked about the reasons for their withdrawal. Further, the participants were monitored for serious adverse events, such as falls or physical illness, in each intervention program as well as problems associated with the implementation of the online program. After the intervention, the participants were also asked for their opinions about the program in a free-writing format.

### Measures

All participants were evaluated using an online questionnaire at the time of their participation in the program (pre-intervention) and at the end of the program (post-intervention). The questions were related to the (1) participants' characteristics, (2) frailty screening, (3) fear of falling, (4) confidence in walking, and (5) subjective health indices. In addition, daily steps were measured.

#### Participants' Characteristics

Before the intervention, the participants' baseline information such as age, sex, medical history, height, and weight were obtained.

#### Frailty Screening Index

The validated Frailty Screening Index (Yamada & Arai, 2015) was used to classify the participants' frailty status. The Frailty Screening Index consists of simple yes or no questions related to the following five items: nutrition/shrinking, physical function, physical activity, forgetfulness, and emotions/exhaustion. Following the developers' classification, a score of 3 or higher was classified as frail, 1–2 as pre-frail, and 0 as robust.

#### Fear of Falling

The modified Falls Efficacy Scale (m-FES) was used to assess the fear of falling pre- and post-intervention (Hill et al., 1996). The m-FES is an assessment scale suitable for community-dwelling older adults because the questionnaire not only examines the ability to perform basic activities of daily living (ADL) such as bathing, which was included in the original FES, but also the ability to perform instrumental ADLs such as shopping. The questionnaire consisted of 14 items, and participants were asked to rate their confidence in their ability to perform each item on a scale of 0–10. Lower scores indicated a higher fear of falling. The validity and reliability of the m-FES were confirmed by Hill et al. (1996) and the Japanese version prepared by Kondo et al. (1999).

#### Confidence in Walking

The modified Gait Efficacy Scale (m-GES) was used to assess the participants' confidence in walking before and after the intervention (Newell et al., 2012). The m-GES is an index that is based upon assumptions appropriate for everyday living environments, such as grass or surfaces with obstacles such as steps. The questionnaire is comprised of 10 items. Participants were instructed to rate their confidence in their ability to walk safely on a scale of 1–10. Higher scores indicated higher confidence in walking. The m-GES was deemed validated and reliable by Newell et al. (2012) and the Japanese version was validated by Makizako et al. (2013).

#### Self-rated Health

Self-rated health was assessed pre- and post-intervention. The participants were asked to rate their health status on a 4-point scale: 4 as very healthy, 3 as fair health, 2 as not very healthy, and 1 as not healthy. This question was formulated based on the method used in a previous study by Kera et al. (2021).

#### Daily Steps

The participants measured the number of steps each day. They were asked to use the pedometer application initially installed on their smartphones. These included Google Fit (Google LCC, USA) on Android devices and Health Care (Apple Inc., USA) on iPhone devices. They were also asked to carry their smartphones from the time they woke up until bedtime. Those who had difficulty measuring the number of steps using their smartphones were provided with pedometers. At 9:00 p.m., a reminder to report the daily number of steps was sent to the LINE group, and all participants self-reported this information via message before bedtime. The average number of daily steps taken during the first seven days and the last seven days of the intervention were used in the analysis. We used the methodology of a similar previous study examining changes in daily step counts following an exercise intervention for community-dwelling older adults (Dohrn et al., 2017; Uchida et al., 2020).

#### Statistical Analysis

The participants' characteristics were analyzed using descriptive statistics. The withdrawal rate was calculated as follows: the number of intervention completers/number of participants who gave their consent to participate × 100. The individual attendance rate was calculated as the number of individual attendances/8 times × 100, while the overall attendance rate was calculated as the total number of attendances of intervention completers/(8 times × number of intervention completers) × 100. Intervention completers were defined as those who had not requested to withdraw from the program by the end of the intervention. To compare the m-FES score, m-GES score, and daily steps pre- and post-intervention, a paired t-test was used for variables with normal distribution or Wilcoxon's signed-rank sum test for those with non-normal distribution. Self-rated health was grouped into two groups, one group that maintained/improved and the other that decreased after the intervention, by comparing the pre- and post-intervention responses. This is a common grouping strategy used in previous studies (Barenfeld et al., 2018; Behm et al., 2014). A chi-square test was used to compare the difference in percentages between the maintained/improved group and the decreased group. The analysis was two tailed with a statistical significance level of 5%. Statistical analysis was performed using the JMP Pro statistical software (version 16.1; SAS Institute Japan Co., Tokyo, Japan).

## Results

### Monitoring of Program Implementation

A total of 25 individuals agreed to participate in the study. Four participants withdrew from the study due to health or family reasons. Thus, the withdrawal rate from the intervention was 16.0%, and the individual attendance rates were 62.5% – 100%, and the overall attendance rate was 94.05%. None of the study participants experienced serious adverse events such as falls or physical illness during the intervention program.

Three participants experienced delays and disruptions in the audio and video due to poor home telecommunication environments. Two of the three were able to restore their connection by reconfiguring their WiFi, while one continued to participate despite the persistent wireless network issues. Other participants occasionally experienced poor communication, but it was temporary and not a major problem.

### Participants' Characteristics

One participant did not respond to the post-intervention questionnaire. Thus, only 20 participants were included in the final analysis. The participants' characteristics are presented in Table 1. The mean age was 76.9 ± 5.7 years; nine were male and 11 were female. In terms of frailty status, seven participants were classified as robust, nine as pre-frail, and four as frail.

**Table 1:** Participant Characteristics

Variables		N=20
Sex	Male	9 (45.0)
	Female	11 (55.0)
Age		76.9 ± 5.7
BMI (kg/cm^2^)		22.5 ± 3.3
Frailty	Robust	7 (35.0)
	Pre-frail	9 (45.0)
	Frail	4 (20.0)

N (%) for sex and frail

Mean±SD for age and BMI

### Pre-intervention and Post-intervention Comparisons

The m-FES and m-GES and daily steps were all non-normally distributed. Wilcoxon's signed-rank sum test was performed to compare the m-FES score, m-GES score, and daily steps pre-intervention and post-intervention. Results, presented in Table 2, showed that post-intervention m-GES and daily steps were significantly greater. No significant differences were found in the m-FES score. Results of the chi-square test showed that self-rated health was significantly greater in the maintained/improved group.

**Table 2:** Results of Pre- and Post-intervention Comparisons

Variables	Pre	Post	p-value
m-FES	130.45±20.4	132.85±18.0	.131
m-GES	87.9±20.3	90.7±18.7	.030^*^
Daily steps	6881±2347	7501±3020	.022^*^
Self-rated health	Maintained/improved	17 (85.0)	.002^*^
	decreased	3 (15.0)	

Wilcoxon signed-rank sum test for m-FES, m-GES, and daily steps (mean ± SD)

Chi-Square test for self-rated health (N (%))

* p<0.05

### Participants' Response

Following the intervention, participants were asked to provide their opinions regarding the program in a free-writing format. Some of the positive comments were as follows: “It was just as good online as in-person classes,” “It was nice to participate from home,” and “I learned how to use my smart phone.” Conversely, three respondents commented negatively due to poor connection: “It was hard to hear the sound.”

## Discussion

This study aimed to (1) examine the feasibility of an online interactive exercise class for community-dwelling older adults and (2) preliminarily examine changes in physical activity and self-efficacy. Throughout the online interactive exercise class, none of the participants experienced serious adverse events, and only minor problems associated with the online implementation of the intervention occurred. Additionally, the responses from participants were positive. The Wilcoxon rank-sum test results showed significant improvements in the m-GES score and daily steps pre-intervention and post-intervention. Furthermore, self-rated health was significantly greater in the maintained/improved group, based on the result of the chisquare test.

The average age of the participants was 76.9 years, approximately the same as that in previous studies of community-dwelling older adults (Nishiguch et al., 2015; Suzuki et al., 2004). Regarding frailty status, the proportion of participants in the robust group was similar to, that in the pre-frail group was lower than, and that in the frail group was higher than those in a previous study conducted in Japan using the same index (Yamada et al., 2015). Thus, the participants in this study were more likely to be frail than the general community-dwelling older population.

A review of intervention studies on fall prevention in the community-dwelling older population reported a withdrawal rate of 9.1%–16% and a participation rate of over 80% after a 2–3-month intervention period (Nyman et al., 2012). Moreover, a previous study conducting online interactive exercise sessions for adults aged 25–58 years reported a withdrawal rate of 50% (Santabarbara et al., 2022). An online intervention study of cancer patients an average age of 65 years, reported participation rates of 80% for one-on-one online sessions and 29% for group-based programs (Dennett et al. 2021). In this study, the withdrawal and participation rates were comparable to those of intervention studies conducted in community-dwelling older individuals, which is considered to be a good rate for online interventions. Additionally, no serious problems such as falls or physical illnesses occurred during the intervention period. Therefore, the program could be conducted safely. A few participants experienced poor communication conditions owing to audio and video problems. However, in most cases, the situation improved by simple on-the-spot operations and did not pose a major problem. During the implementation stage, these technical problems were considered as issues that can be resolved by preparing the communication environment in advance. When participants provided free-writing comments about the program, the majority were positive. Based on the above findings, an online interactive program for community-dwelling older adults was considered feasible.

Results of pre- and post-intervention comparisons showed that the m-GES scores and daily steps significantly improved after the intervention. Further, the maintained/improved group showed significantly greater post-intervention self-rated health. A previous study reported that an in-person exercise class for community-dwelling older adults significantly improved their self-efficacy for walking, falling, and daily steps (Nishiguch et al., 2015; Suzuki et al., 2004). Furthermore, some participants reported that their self-rated health was maintained or increased after participating in the exercise classes (Barenfeld et al., 2018; Behm et al., 2014). In this study, the exercise class was conducted in an online setting, and the results were similar to those obtained in-person. However, findings of reviews on step counts showed that continuous monitoring improved the step counts (Chaudhry et al., 2020). Therefore, the daily reporting of step count might have contributed to the improvement in the step count in this study, and this intervention should be closely examined.

The m-FES score did not improve. In this study, the mean m-FES score was 130.5 pre-intervention. A previous study that measured the m-FES score in community-dwelling older individuals reported a mean value of 113.7–123.1 (Quigley et al., 2014). The highest m-FES score was 140. It is possible that the participants in this study did not show a significant improvement as their baseline m-FES score was high.

This study had several limitations. First, it was a before-and-after study and not a randomized controlled trial. Further investigation of the effectiveness of this intervention is required. Second, the intervention only lasted for a short time. The Ministry of Health, Labour and Welfare recommends that care prevention programs should be conducted for at least three months (Ministry of Health, Labour and Welfare, 2022a). Since this study was only conducted for a month, further study is required to determine the effectiveness of the program when performed over a longer period. In addition, this study required digital devices such as smartphones and computers for all phases. Therefore, a bias arose in that people who did not own or were not accustomed to using digital devices were unable to participate. In the future, it will be necessary to consider lending digital devices and providing training on how to operate them to enable people in a variety of situations to participate. Furthermore, to simplify the recording of daily steps in this study, a self-reporting method was used. It is possible that incorrect daily steps may have been reported. To ensure accuracy, it is necessary to download historical data or directly check the logs.

However, this study showed that online interactive exercise classes can be safely implemented in community-dwelling older adults. Moreover, it may be as effective as in-person exercise classes in improving physical activity and self-efficacy. In today's society, where digital communication is more common, we believe that we were able to demonstrate the possibility that care prevention programs can also be conducted online. In future studies, it will be necessary to consider support for emergency incidents and communication environments in collaboration with local authorities and care facilities in the participants' residential areas to provide safe and seamless interventions for remote areas.

## Conclusion

This study found that online interactive exercise classes for community-dwelling older individuals in Japan were safe and feasible. Moreover, they could improve an older adults' physical activity and self-efficacy. However, further randomized controlled trials are warranted to scrutinize the effectiveness of the intervention and examine the effects of long-term interventions.
